# Single-dose effects of methylphenidate and atomoxetine on functional connectivity during an n-back task in boys with ADHD

**DOI:** 10.1007/s00213-023-06422-7

**Published:** 2023-07-27

**Authors:** Olivia S. Kowalczyk, Ana I. Cubillo, Marion Criaud, Vincent Giampietro, Owen G. O’Daly, Mitul A. Mehta, Katya Rubia

**Affiliations:** 1 Department of Neuroimaging, Institute of Psychiatry, Psychology & Neuroscience, King’s College London, London, UK; 2 Department of Child & Adolescent Psychiatry, Institute of Psychiatry, Psychology & Neuroscience, King’s College London, London, UK; 3 Jacobs Center for Productive Youth Development, Zurich Center for Neuroeconomics, University of Zürich, Zürich, Switzerland

**Keywords:** Attention-deficit/hyperactivity disorder, ADHD, Functional magnetic resonance imaging, fMRI, Working memory, Functional connectivity, Psychophysiological interaction, Methylphenidate, Atomoxetine

## Abstract

**Rationale:**

Working memory deficits and associated neurofunctional abnormalities are frequently reported in attention-deficit/hyperactivity disorder (ADHD). Methylphenidate and atomoxetine improve working memory performance and increase activation of regions under-functioning in ADHD. Additionally, methylphenidate has been observed to modulate functional networks involved in working memory. No research, however, has examined the effects of atomoxetine or compared the two drugs.

**Objectives:**

This study aimed to test methylphenidate and atomoxetine effects on functional connectivity during working memory in boys with ADHD.

**Methods:**

We tested comparative effects of methylphenidate and atomoxetine on functional connectivity during the n-back task in 19 medication-naïve boys with ADHD (10–15 years old) relative to placebo and assessed potential normalisation effects of brain dysfunctions under placebo relative to 20 age-matched neurotypical boys. Patients were scanned in a randomised, double-blind, cross-over design under single doses of methylphenidate, atomoxetine, and placebo. Controls were scanned once, unmedicated.

**Results:**

Patients under placebo showed abnormally increased connectivity between right superior parietal gyrus (rSPG) and left central operculum/insula. This hyperconnectivity was not observed when patients were under methylphenidate or atomoxetine. Furthermore, under methylphenidate, patients showed increased connectivity relative to controls between right middle frontal gyrus (rMFG) and cingulo-temporo-parietal and striato-thalamic regions, and between rSPG and cingulo-parietal areas. Interrogating these networks within patients revealed increased connectivity between both rMFG and rSPG and right supramarginal gyrus under methylphenidate relative to placebo. Nonetheless, no differences across drug conditions were observed within patients at whole brain level. No drug effects on performance were observed.

**Conclusions:**

This study shows shared modulating effects of methylphenidate and atomoxetine on parieto-insular connectivity but exclusive effects of methylphenidate on connectivity increases in fronto-temporo-parietal and fronto-striato-thalamic networks in ADHD.

**Supplementary Information:**

The online version contains supplementary material available at 10.1007/s00213-023-06422-7.

## Introduction

Attention-deficit/hyperactivity disorder (ADHD) is characterised by developmentally inappropriate levels of hyperactivity, impulsivity, and/or inattention (American Psychiatric Association 2013). Working memory deficits (Martinussen et al. 2005; Willcutt et al. 2005; Coghill et al. 2018; Pievsky and McGrath 2018a; Ramos et al. 2019) and their accompanying reductions in fronto-striatal and temporo-parietal activation (Silk et al. 2005; Vance et al. 2007; Kobel et al. 2009; Cortese et al. 2012; Cubillo et al. 2014a; McCarthy et al. 2014; Chantiluke et al. 2015), along with altered patterns of functional connectivity (Wolf et al. 2009; Massat et al. 2012; Bédard et al. 2014; Wu et al. 2017), have been widely reported in ADHD.

Functional magnetic resonance imaging (fMRI) studies of working memory in youth with ADHD relative to neurotypical individuals show consistent increases in fronto-parietal coupling (Massat et al. 2012; Bédard et al. 2014; Wu et al. 2017), along with connectivity increases in networks comprising posterior areas such as cuneus, precuneus, and occipital regions (Wu et al. 2017), between occipital and cortico-striato-cerebellar regions, and between cerebellum and brainstem (Massat et al. 2012). Similarly, adults with ADHD relative to neurotypical adults show connectivity increases in various networks that include inferior and superior frontal regions, dorsal cingulate, and cuneus (Wolf et al. 2009). Additionally, abnormal reductions in working memory-related connectivity have been observed within executive control (Wu et al. 2017) and fronto-cingulate networks in youth (Bédard et al. 2014), and within fronto-cingulo-parieto-cerebellar networks in adults with ADHD (Wolf et al. 2009). This variability in connectivity differences may be further exacerbated by the known functional brain maturation processes taking place in adolescence and the variability of ages of participants recruited for research (Pfeifer and Allen 2021).

The catecholamine transporter blocker methylphenidate (Wilens 2008; Faraone 2018) and the non-stimulant, noradrenaline transporter blocker atomoxetine (Bymaster et al. 2002; Gallezot et al. 2011) are the most commonly used pharmacological treatments for ADHD showing good clinical efficacy (Cortese et al. 2018). However, their specific neurofunctional mechanisms of action are not fully understood. While both methylphenidate and atomoxetine have shown to improve working memory performance of individuals with ADHD (Gau and Shang 2010; Yang et al. 2012; Shang and Gau 2012; Ni et al. 2013; Coghill et al. 2014; Pievsky and McGrath 2018b; Rubio Morell and Expósito 2019), distinct neurofunctional effects of stimulants and non-stimulants have been observed (Cubillo et al. 2014a). A meta-regression of stimulant effects on brain activation in ADHD during n-back working memory paradigms found that treatment was associated with greater activation in middle and superior frontal regions (McCarthy et al. 2014). A direct comparison of single doses of methylphenidate and atomoxetine on brain activation during the n-back task in the same cohort of children with ADHD found both drug-specific and shared effects (Cubillo et al. 2014a). Both drugs increased fronto-insular, temporal, and striatal activation in patients compared to unmedicated controls. However, atomoxetine selectively normalised abnormalities observed under placebo in the right dorsolateral prefrontal cortex (DLPFC) and upregulated this region relative to methylphenidate, while methylphenidate selectively enhanced activity of the left inferior frontal cortex (IFC)/DLPFC relative to controls during the 2-back condition (Cubillo et al. 2014a).

Also, methylphenidate has shown to normalise functional connectivity differences between boys with ADHD and neurotypical controls in executive control, fronto-parietal, and auditory networks, and to increase the connectivity within the executive control network compared to placebo (Wu et al. 2017). Studies further showed that, in youth with ADHD, stimulants decrease fronto-striatal connectivity compared to no medication (Sheridan et al. 2010) and increase the connectivity within the fronto-parietal network, as well as this network’s functional connections with other cortical regions, including anterior cingulate cortex (ACC), ventrolateral prefrontal cortex, and precuneus compared to placebo (Wong and Stevens 2012). Nonetheless, no studies explored the effects of atomoxetine on working memory-related connectivity or compared the network effects of the two drugs in ADHD. Given that adolescence is a period of progressive neurofunctional maturation and specialisation of brain networks (Rubia 2013; Pfeifer and Allen 2021), it is crucial to understand the effects medications may have during this developmental stage.

Consequently, the aim of this pseudo-randomised, double-blind, placebo-controlled, cross-over fMRI study was to test the comparative effects of single doses of methylphenidate and atomoxetine relative to placebo on functional networks involved in working memory in medication-naïve boys with ADHD, and to test for potential normalisation effects of the two drugs on brain connectivity differences during placebo relative to unmedicated age-matched neurotypical controls. This study extends a previously published analysis of shared and distinct effects of the two drugs on brain activation during working memory in the same cohort (Cubillo et al. 2014a) to the network level. The advantage of single-dose comparisons is studying drug effects and probing the role of catecholamines without the confounds of symptomatic improvement, side effects, or brain adaptation following long-term dosing (Konrad et al. 2007; Nakao et al. 2011; Fusar-Poli et al. 2012; Frodl and Skokauskas 2012; McCarthy et al. 2014; Lukito et al. 2020).

In line with previous reports of fronto-parietal hyperconnectivity during working memory (Massat et al. 2012; Bédard et al. 2014; Wu et al. 2017), we expected greater frontal and parietal connectivity during working memory compared to the control task condition in individuals with ADHD under placebo relative to controls. Considering evidence of normalisation/upregulation effects of methylphenidate and atomoxetine on working memory-related brain activation (Cubillo et al. 2014a; McCarthy et al. 2014) and of normalising/modulating effects of methylphenidate on working memory-related functional connectivity (Sheridan et al. 2010; Wong and Stevens 2012; Wu et al. 2017), we expected that both medications would minimise the connectivity differences between patients and controls.

## Methods

The analysis of this data focusing on brain activation has been published previously (Cubillo et al. 2014a). The analysis described here focuses on working memory-related functional connectivity.

### Participants

Data from 19 medication-naïve boys (10–15 years old) with a diagnosis of ADHD combined presentation and 20 neurotypical control boys (10–15 years old) were included in this study. Patients were recruited from South London Child and Adolescent Mental Health Services. ADHD diagnosis was confirmed by an experienced child psychiatrist using the standardised Maudsley Diagnostic Interview (DSM-IV-TR criteria; Goldberg and Murray 2002). Boys with ADHD scored above the clinical threshold for ADHD symptoms on the strengths and difficulties questionnaire for parents (SDQ; Goodman and Scott 1999) and the Conners parent rating scale (CPRS-R; Conners 2008), and below the clinical threshold on the social communication questionnaire (SCQ) to exclude participants with high autism traits (Rutter et al. 2003). Where SCQ scores were inconclusive or not provided, the child’s clinician was consulted to rule out autism comorbidity. Neurotypical boys were recruited through advertisement in the same South London area. They scored below the clinical cut-off on the SDQ, SCQ, and CPRS-R (Table 1). All participants were right-handed.

**Table 1:** Sociodemographic characteristics of neurotypical controls and patients with ADHD

Variable	Control (*N* = 20)		ADHD (*N* = 19)		Group Comparison
	Mean	SD	Mean	SD	
Age [months]	152.1	20.18	157.42	19.07	*t*(37) = 0.84, *p* = 0.404, Hedges’ *g* = 0.26, 95% CI [−0.37; 0.9]
IQ	110.8	13.12	91.21	11.35	*t*(37) = 4.99, *p* < 0.001, Hedges’ *g* = −1.56, 95% CI [−2.29; -0.84]
SDQ	3.9	3.71	22.32	6.05	*t*(30) = 11.39, *p* < 0.001, Hedges’ *g* = 3.62, 95% CI [2.59; 4.65]
SCQ	1.15	2.18	9.84	4.06	*t*(27) = 8.27, *p* < 0.001, Hedges’ *g* = 2.63, 95% CI [1.76; 3.5]

*ADHD,* Attention-Deficit/Hyperactivity Disorder; *IQ*, Intelligence Quotient; *SCQ*, Social Communication Questionnaire; *SDQ*, Strengths and Difficulties Questionnaire

Exclusion criteria for all participants were MRI-related contraindications, mean framewise displacement during scanning >1mm, IQ <70 on the Wechsler abbreviated scale of intelligence (WASI; Wechsler 1999), history of substance abuse, neurological deficits, presence of psychiatric disorders (except for conduct disorder/oppositional defiant disorder in the ADHD group, *N* = 2), learning disability, reading, speech, or language disorder. Data for additional 11 participants were available (*N*_ADHD_ = 1, *N*_control_ = 10) but were not included in the current study due to excessive motion (*N*_ADHD_ = 1), poor functional data normalisation (*N*_control_ = 1), and control participant’s age exceeding the age range of patients (*N*_control_ = 9).

Welch’s *t*-tests showed no significant group difference for age but a significant difference for IQ with ADHD participants scoring lower than controls, which is typical in this population (Rommel et al. 2015). Moreover, ADHD participants scored significantly higher on the SDQ and SCQ (Table 1). Missing data points (*N*_SCQ_ = 9, *N*_SDQ_ = 2) were mean imputed.

Participants were reimbursed £50 for each session. Parental/child written informed consent/assent were obtained. Ethical approval was granted by the Joint South London and Maudsley/Institute of Psychiatry, Psychology & Neuroscience Research Ethics Committee (07/H0807/84).

### Procedure

Boys with ADHD were scanned in a double-blind, placebo-controlled, cross-over design. On each scanning session (1 week apart), they received a single dose of either placebo (vitamin C, 50mg), methylphenidate (Equasym, 0.3mg/kg, 5–20mg), or atomoxetine (Strattera, 1mg/kg, 16–66mg) in a pseudo-randomised order. Dosages were determined following National Institute for Health and Care Excellence guidelines at the time of the study (National Institute for Health and Care Excellence 2008). Both drugs and placebo were administered in identical capsules 1.5 h before scanning to allow maximum absorption (Chan et al. 1983; Witcher et al. 2003) and to preserve blinding. Similar dosages and time lapses between drug administration and scans have shown to be sufficient to observe changes in brain activation and performance in ADHD (Cubillo et al. 2014a, 2014b; Kowalczyk et al. 2019; Rubia et al. 2011a, 2011b; Smith et al. 2013).

Controls were scanned once, unmedicated.

### N-back paradigm

**Fig. 1:**
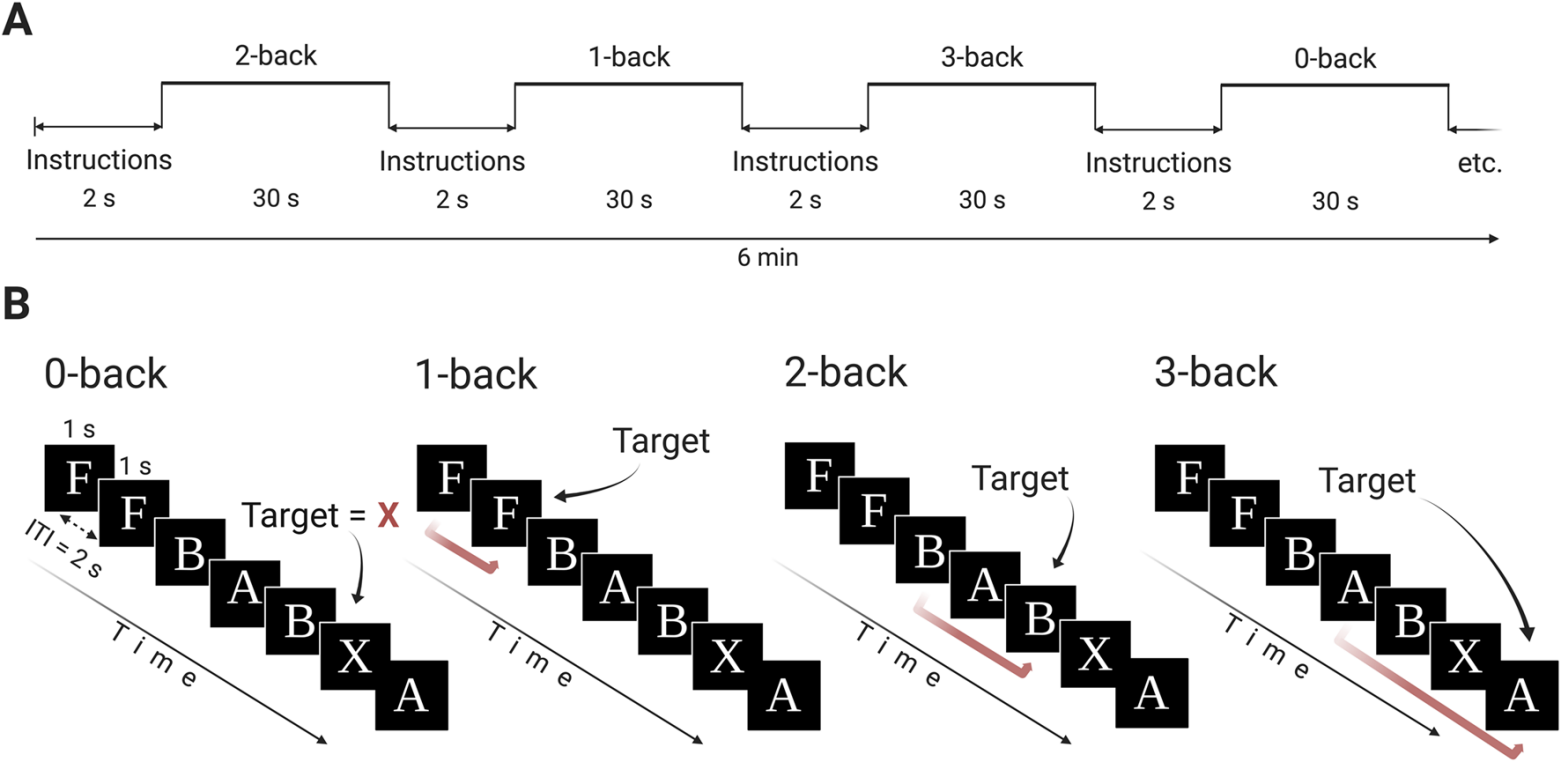
A schematic representation of the n-back block design task. **A**. A visual representation of the task block structure. **B**. Examples of n-back conditions of different working memory load (0-, 1-, 2-, and 3-back). ITI, inter-trial interval

The 6-min block design working memory task consisted of four conditions (Ginestet and Simmons 2011; Cubillo et al. 2014a). This parametric task with increasing working memory load was chosen to avoid floor or ceiling effects in task performance (Pongpipat et al. 2021). During 1-back, 2-back, and 3-back conditions, participants were presented with series of letters (1s duration, 2s inter-trial interval) and responded with their right thumb using a button box whenever the letter shown was the same as the letter presented one, two, or three before it, respectively (e.g. 2-back: B/**J**/A/**J**). This requires both storage and continuous updating of stimuli held in working memory (Rac-Lubashevsky and Kessler 2016). In the baseline vigilance 0-back condition, participants responded to each ‘X’ that appeared on the screen (Fig. 1). The task consisted of 180 trials presented in 12 blocks. Before each block, written instructions (2s) indicated which condition would be shown. Each block comprised one condition lasting 30s and consisting of 14 stimuli presentations: 3 targets and 11 non-targets. Each condition was presented three times. Performance data were recorded during scanning.

Participants practised the task once before scanning.

### MRI acquisition

Data were acquired on a GE Signa HDx 3T system (General Electric, Milwaukee, Wisconsin) with an 8-channel head coil at the Centre for Neuroimaging Sciences, King’s College London. Functional T2*-weighted data comprised 39 interleaved slices acquired with a gradient EPI sequence bottom-to-top over 186 volumes depicting BOLD contrast covering the whole brain (TE = 30ms, TR = 2s, flip angle = 75°, in-plane voxel size = 3.75mm, slice thickness = 3.5mm, slice gap = 0.5mm). Whole-head structural image was acquired at the beginning of each scanning session using an MPRAGE protocol using parameters based on ADNI (TE = 2.85ms, TR = 6.99s, flip angle = 8°, in-plane voxel size = 1.02mm, 166 slices, slice thickness = 1.2mm, slice gap = 1.2mm).

### Performance data analysis

Dependent variables were percentage accuracy in identifying targets, mean reaction time (MRT), and intra-subject variability of reaction time indexed by standard deviation of reaction time (SDRT). Data were analysed using R (v4.0.2; R Core Team 2017) with rstatix (v0.6.0; Kassambara 2020), effsize (v0.8.0; Torchiano 2020), stats (v4.0.2; R Core Team 2017), and tidyverse (v1.3.0; Wickham and RStudio 2019) packages. Performance within patients was investigated using a repeated measures ANOVA with drug condition (placebo, methylphenidate, atomoxetine) and working memory load (0-back, 1-back, 2-back, and 3-back) as within-subject factors. For case-control comparisons, three mixed-measures ANOVAs compared participants with ADHD under each drug condition with controls. Working memory load was a within-subject factor and group (ADHD or control) a between-subject factor.

### fMRI data analysis

#### Data preprocessing

Data were processed using SPM12 (v7487; https://fil.ion.ucl.ac.uk/spm; Penny et al. 2007) and MATLAB (v9.5.0; MATLAB 2018). The origin of all images was reset to the anterior commissure. Each participant’s functional data were slice-time corrected, realigned, and co-registered with the participant’s anatomical image, and smoothed at 6mm FWHM Gaussian kernel. Anatomical images were segmented into grey matter, white matter, cerebrospinal fluid, and non-brain components using unified segmentation. The resultant unsampled grey and white matter images were used to create a study-specific template using DARTEL (Ashburner 2007). The generated deformation flow fields were used in functional data normalisation to the MNI template. Visual quality checks were performed at all stages.

#### Seed region selection

Three seed regions were chosen for functional connectivity analysis based on their consistent responses to working memory paradigms in paediatric and adult meta-analyses (Owen et al. 2005; Andre et al. 2015; Yaple and Arsalidou 2018) and in the analysis of brain activation in our control group during high working memory load compared to baseline (2-back > 0-back; Supplementary Materials), including right superior parietal gyrus (rSPG; MNI coordinates [*x*, *y*, *z*]: 23, −66, 46; Yaple and Arsalidou 2018), right middle frontal gyrus (rMFG; 31, −1, 56; Andre et al. 2015), and right DLPFC (rDLPFC; 43, 31, 30; Owen et al. 2005). The seeds were created as 5mm radius spheres using MarsBar (Brett et al. 2002).

#### Generalised psychophysiological interaction

Functional connectivity during the n-back task was examined using generalised psychophysiological interaction (gPPI) method (McLaren et al. 2012). Separate gPPI models were created for each seed region. For each model, the first eigenvariate of the seed’s time-series was extracted for each participant and deconvolved to obtain an estimate of the physiological activity within that region. Subsequently, five PPI regressors were created by multiplying the estimate of the seed's physiological activity by vectors representing each of the task conditions (instruction, 0-back, 1-back, 2-back, and 3-back). PPI regressors were convolved with a canonical haemodynamic response function and entered into a GLM together with a regressor corresponding to the seed’s time-series, 24 motion parameters (Friston et al. 1996), scrubbing regressors for volumes where framewise displacement (FD) > 0.5mm (Power et al. 2012, 2014), and two nuisance regressors modelling mean white matter and cerebrospinal fluid signal. The 3-back condition was not included in contrasts, as participants in all groups were observed to make errors on >30% of trials. The contrast of interest explored the interaction of the seed with high working memory load condition compared to the baseline vigilance condition (2-back > 0-back). This contrast from first-level models was taken forward to group-level random-effects analysis.

To check the gPPI modelling quality, three one-sample *t*-tests of the interaction between the high working memory load contrast (2-back > 0-back) and each of the seeds were conducted in the control group (Supplementary Materials).

For hypothesis-testing, independent samples *t*-tests with age in months as a covariate were used to compare participants with ADHD under each drug condition with the neurotypical control group. Paired samples *t*-tests explored drug effects within the ADHD cohort. Cluster-based FWE correction (*p* < 0.05) with a cluster-forming threshold of *p*_uncorrected_ < 0.001 was applied to account for multiple comparisons.

#### Conjunction analyses for the assessment of normalisation and upregulation effects

The goal of treatments in psychiatry is to minimise dysfunction observed in patients, potentially through *normalisation* of function. An absence of a significant difference, however, cannot be considered as evidence of normalisation (Ranganathan et al. 2015). A conjunction analysis between a comparison of neurotypical controls relative to patients under placebo *and* patients under a drug relative to patients under placebo would indicate regions where shared activation or connectivity in patients under a drug and neurotypical controls suggests a drug-related normalisation. Consequently, to test for potential normalisation effects of drugs on ADHD-related neurofunctional abnormalities and any drug-related compensatory changes, we used confirmatory conjunction analyses.

### Sensitivity analysis

Sensitivity analyses were conducted on performance and fMRI data excluding participants with comorbid oppositional defiant and/or conduct disorder (*N* = 2).

### Scan order effects

Repeated measures ANOVAs were used to test for scan order effects in patients using accuracy, MRT, and SDRT.

## Results

### Task performance

Descriptive statistics for performance are reported in Table 2.

**Table 2:** Performance measures and analyses for the n-back task for neurotypical controls and patients with ADHD under each drug condition

Task condition	Control (*N* = 20)		ADHD PLA (*N* = 19)		ADHD MPH (*N* = 19)		ADHD ATX (*N* = 19)		Comparison	Analysis		
	Mean	SD	Mean	SD	Mean	SD	Mean	SD		Group effect	Task load effect	Interaction effect
Accuracy [%]												
0-back	96.15	10.87	98.26	4.12	98.84	3.47	97.11	8.07	Within ADHD	*F*(2, 36) = 1.44, *p* = 0.251, *η*^*2*^ = 0.01	*F*(1.55, 27.98) = 30.4, *p* < 0.001, *η*^*2*^ = 0.36**	*F*(3.94, 70.91) = 0.78, *p* = 0.542, *η*^*2*^ = 0.01
1-back	93.35	15.48	97.11	6.18	97.68	5.89	94.74	15.93	ADHD PLA vs. control	*F*(1, 37) = 0.37, *p* = 0.54, *η*^*2*^ = 0.01	*F*(1.85, 68.62) = 51.11, *p* < 0.001, *η*^*2*^ = 0.35**	*F*(1.85, 68.62) = 2.4, *p* = 0.102, *η*^*2*^ = 0.3
2-back	86.15	22.52	76.63	26.52	84.9	20.98	82.53	25.75	ADHD MPH vs control	*F*(1, 37) = 0.12, *p* = 0.726, *η*^*2*^ < 0.01	*F*(2.22, 82.24) = 45.65, *p* < 0.001, *η*^*2*^ = 0.34**	*F*(2.22, 82.24) = 0.39, *p* = 0.703, *η*^*2*^ < 0.01
3-back	67.4	18.99	59.74	28.84	67.42	23.65	65.53	25.24	ADHD ATX vs. control	*F*(1, 37) = 0.03, *p* = 0.868, *η*^*2*^ < 0.01	*F*(2.24, 83.01) = 39.84, *p* < 0.001, *η*^*2*^ = 0.29**	*F*(2.24, 83.01) = 0.3, *p* = 0.765, *η*^*2*^ < 0.01
Mean reaction time [ms]												
0-back	499.16	80.66	516.12	101.19	507.68	101.68	516.16	96.42	Within ADHD	*F*(2, 36) = 0.23, *p* = 0.797, *η*^*2*^ < 0.01	*F*(3, 54) = 48.24, *p* < 0.001, *η*^*2*^ = 0.23**	*F*(3.88, 69.84) = 0.73, *p* = 0.57, *η*^*2*^ = 0.01
1-back	612.17	154.29	607	152.12	597.6	98.52	599.75	154.44	ADHD PLA vs. control	*F*(1, 37) = 0.07, *p* = 0.793, *η*^*2*^ < 0.01	*F*(3, 111) = 39.48, *p* < 0.001, *η*^*2*^ = 0.25**	*F*(3, 111) = 3.2, *p* = 0.026, *η*^*2*^ = 0.03*
2-back	638.15	132.56	671.84	176.28	685.74	154.28	708.37	212.57	ADHD MPH vs control	*F*(1, 37) = 0.03, *p* = 0.867, *η*^*2*^ < 0.01	*F*(2.31, 85.51) = 38.4, *p* < 0.001, *η*^*2*^ = 0.32**	*F*(2.31, 85.51) = 0.75, *p* = 0.493, *η*^*2*^ = 0.01
3-back	772.4	168.52	686.69	154.33	753.83	204.37	719.04	168.66	ADHD ATX vs. control	*F*(1, 37) = 0.02, *p* = 0.891, *η*^*2*^ < 0.01	*F*(3, 111) = 37.34, *p* < 0.001, *η*^*2*^ = 0.26**	*F*(3, 111) = 2.46, *p* = 0.066, *η*^*2*^ = 0.02
Standard deviation of reaction time [ms]												
0-back	112.3	104.41	122.2	100.18	122.82	112.6	96.68	43.75	Within ADHD	*F*(2, 36) = 0.19, *p* = 0.83, *η*^*2*^ < 0.01	*F*(3, 54) = 24.63, *p* < 0.001, *η*^*2*^ = 0.17**	*F*(6, 108) = 0.49, *p* = 818, *η*^*2*^ = 0.01
1-back	148.32	95.56	189.08	102.59	152.34	48.96	152.28	77.76	ADHD PLA vs. control	*F*(1, 37) = 0.08, *p* = 0.775, *η*^*2*^ < 0.01	*F*(3, 111) = 12.13, *p* < 0.001, *η*^*2*^ = 0.15**	*F*(3, 111) = 0.99, *p* = 0.4, *η*^*2*^ = 0.02
2-back	194.01	104.98	201.78	124.14	205.38	95.85	211.1	122.97	ADHD MPH vs. control	*F*(1, 37) < 0.01, *p* = 0.997, *η*^*2*^ < 0.01	*F*(3, 111) = 15.04, *p* < 0.001, *η*^*2*^ = 0.21**	*F*(3, 111) = 0.37, *p* = 0.774, *η*^*2*^ = 0.01
3-back	256.95	91.3	225.24	138.31	231.28	104.34	241.51	94.29	ADHD ATX vs. control	*F*(1, 37) = 0.02, *p* = 0.889, *η*^*2*^ < 0.01	*F*(3, 111) = 19.68, *p* < 0.001, *η*^*2*^ = 0.258**	*F*(3, 111) = 0.32, *p* = 0.812, *η*^*2*^ = 0.01

*ADHD,* Attention-Ddeficit/Hyperactivity Disorder; *ATX,* Atomoxetine; *MPH*, Methylphenidate; *PLA,* Placebo; *SD,* Standard Deviation

*p < 0.05, **p < 0.001

#### Within-patients comparisons

Repeated measures ANOVAs revealed a significant effect of working memory load on accuracy, MRT, and SDRT. Increasing working memory load was associated with lower accuracy, along with slower and more variable responses across drug conditions. No effects of drug condition or interaction effects between drug condition and working memory load were observed (Table 2).

#### Case-control comparisons

Across all participants, mixed-measures ANOVAs showed that increasing working memory load was associated with reduced accuracy, longer MRT, and larger SDRT. Significant interaction effects between working memory load and group were observed for MRT when controls were compared to patients under placebo, due to patients responding slower during 0-back and 2-back, and faster during 1-back and 3-back; however, post hoc tests revealed these effects were statistically non-significant (0-back, *p* = 0.568; 1-back, *p* = 0.917; 2-back, *p* = 0.506; 3-back, *p* = 0.106). No other statistically significant effects were observed (Table 2). Covarying for age did not affect the results.

### fMRI results

#### Motion

FD was calculated according to Power et al. (2012, 2014) using custom code (https://version.aalto.fi/gitlab/BML/bramila). One-way ANOVA showed no significant group difference between controls and patients under each drug condition in mean FD (*F*(3, 73) = 1.14, *p* = 0.338, *ƞ* ^*2*^= 0.04; control, mean = 0.18mm, SD = 0.08; ADHD_placebo_, mean = 0.22mm, SD = 0.19; ADHD_methylphenidate_, mean = 0.17mm, SD = 0.08; ADHD_atomoxetine_, mean = 0.23mm, SD = 0.12).

#### Within-patients comparisons

Paired samples *t*-tests showed no significant differences for any seeds in within-patients comparisons.

#### Case-control comparisons

**Fig. 2:**
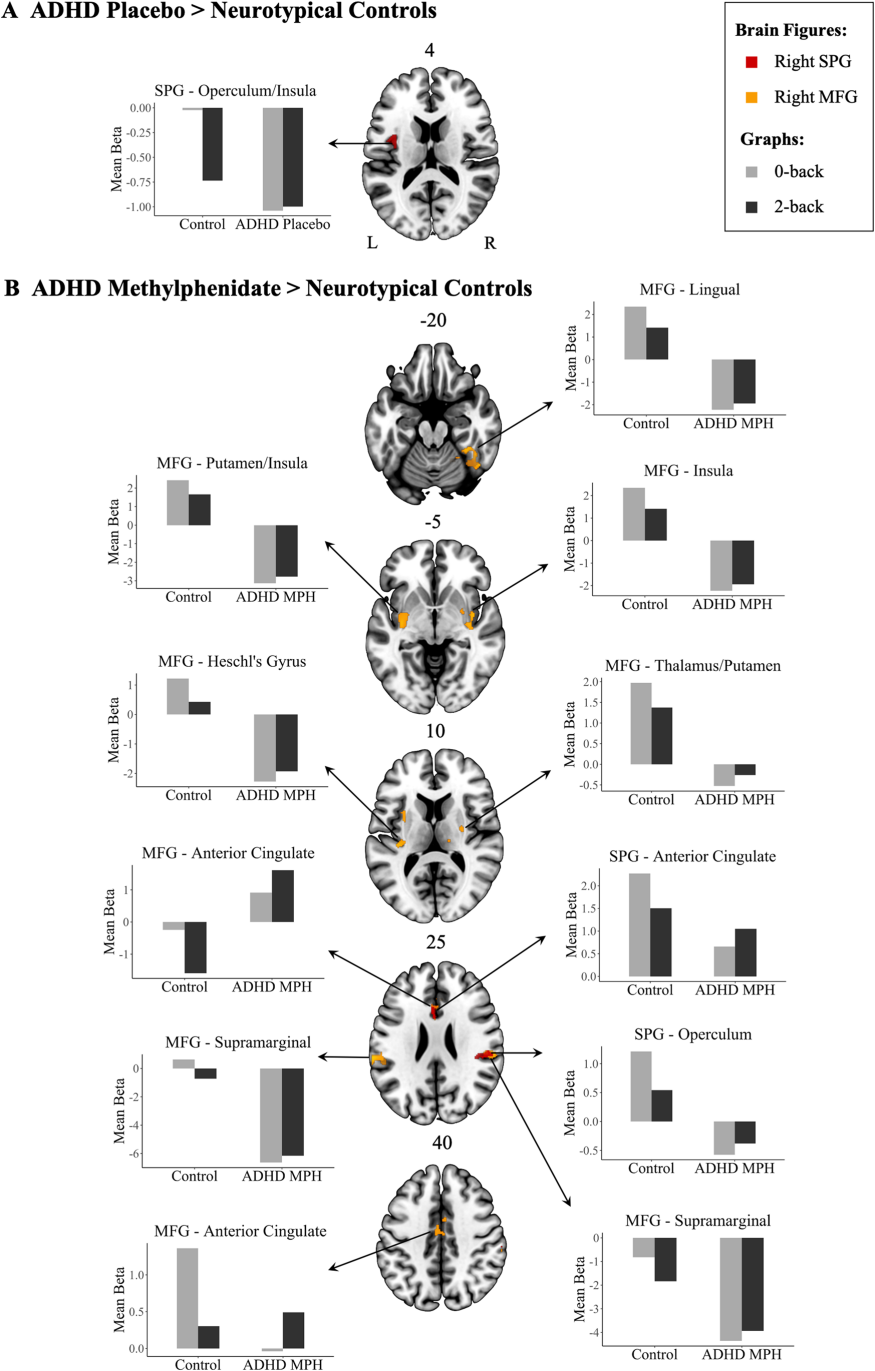
Functional connectivity during 2-back > 0-back and graphs showing mean beta values within each statistically significant cluster during 0-back and 2-back in neurotypical controls and participants with ADHD. **A.** Regions showing significantly higher functional connectivity with the rSPG in participants with ADHD under placebo compared to neurotypical controls. **B.** Regions of greater functional connectivity with rSPG (red) and rMFG (yellow) in the ADHD group under methylphenidate compared to controls. Axial slices are marked with the *z* coordinate. rMFG, right middle frontal gyrus; MPH, methylphenidate; rSPG, right superior parietal gyrus

Under placebo, independent samples *t*-test showed that participants with ADHD exhibited greater connectivity between rSPG and left-hemispheric insula/central operculum relative to controls. This effect was due to more negative beta values during 2-back compared to 0-back in controls, but no such difference in participants with ADHD under placebo (Fig. 2, Table 3). No differences between controls and participants with ADHD under placebo were observed for connectivity with rMFG or rDLPFC.

**Table 3:** Significant clusters of differences between neurotypical controls and participants with ADHD under each drug condition and their peak connectivity during the 2-back > 0-back contrast

Seed	Cluster	Cerebral region	Cluster size (voxels)	Peak MNI coordinate			Peak *z*	Cluster *p*_FWE_
				*x*	*y*	*z*		
ADHD placebo > neurotypical controls								
rSPG	1	L central operculum/insula	483	−36	0	15	4.05	0.021
		L insula		−38	−6	−3	3.89	
		L insula		−34	−9	14	3.8	
ADHD methylphenidate > neurotypical controls								
rMFG	1	R lingual	1278	20	−57	−14	4.18	<0.001
		R temporal occipital fusiform		44	−48	−24	4.15	
		R temporal occipital fusiform		34	−42	−21	3.93	
	2	R insula	1161	36	−14	−8	4.3	<0.001
		R insula		40	3	−2	4.26	
		R Heschl’s gyrus/planum polare		42	−22	3	3.87	
	3	L Heschl’s gyrus/insula	735	−34	−26	9	4.03	0.004
		L white matter/insula		−30	−33	4	3.95	
		L white matter/putamen		−33	−14	−4	3.89	
	4	L supramarginal/postcentral	723	−64	−30	22	4.54	0.004
		L planum temporale/operculum		−63	−26	15	4.36	
		L parietal operculum		−52	−34	24	3.78	
	5	R supramarginal	645	64	−28	34	4.45	0.007
		R parietal operculum		56	−26	24	4.44	
		R parietal operculum/supramarginal		54	−32	33	4.13	
	6	L putamen	571	−27	−3	6	4.26	0.013
		L insula		−32	4	4	3.98	
		L insula		−30	10	12	3.68	
	7	L anterior cingulate	532	−2	24	32	3.69	0.018
		L anterior cingulate		−2	15	22	3.66	
		R anterior cingulate/paracingulate		8	28	27	3.29	
	8	R white matter/thalamus	482	26	−24	4	4.21	0.026
		R thalamus		21	−32	0	3.74	
		R putamen		28	−9	10	3.53	
	9	L anterior cingulate	465	−2	−10	38	3.64	0.03
		L white matter		−14	−20	33	3.6	
		R anterior cingulate		4	2	39	3.41	
rSPG	1	R parietal operculum/supramarginal	581	52	−32	30	4	0.009
		R parietal operculum		42	−28	24	3.94	
		R supramarginal		63	−26	38	3.68	
	2	L paracingulate/anterior cingulate	556	−12	14	34	4.58	0.011
		L anterior cingulate		−2	12	26	3.63	
		R anterior cingulate		2	21	33	3.51	

*ADHD,* Attention-Deficit/Hyperactivity Disorder; *FWE,* Family-Wise Error; *L*, Left; *rMFG,* Right Middle Frontal Gyrus; *MNI*, Montreal Neurological Institute*; R*, Right; *rSPG,* Right Superior Parietal Gyrus

Under methylphenidate, participants with ADHD showed greater connectivity than controls between rMFG and (1) bilateral: insula, ACC, Heschl’s gyrus, putamen, supramarginal gyri (SMG), parietal operculum; (2) left: postcentral gyrus and planum temporale/operculum; (3) right: thalamus, paracingulate gyrus, and temporal occipital fusiform cortex. Additionally, greater connectivity in participants with ADHD under methylphenidate between rSPG and bilateral ACC, as well as right parietal operculum/SMG was found compared to controls. Mean beta values indicated that neurotypical controls exhibited lower beta values during 2-back than 0-back. In contrast, participants with ADHD under methylphenidate showed the opposite effect, with higher beta values during 2-back compared to 0-back (Fig. 2, Table 3). No differences, however, were observed for rDLPFC connectivity between participants with ADHD under methylphenidate and controls.

Comparisons of participants with ADHD under atomoxetine and controls revealed no significant differences for connectivity with any seed.

To test for potential associations between connectivity and performance, 2-back accuracy (main index of performance in this task) was entered into group-level models showing case-control differences as a covariate of interest. Small volume correction was applied to restrict the test to regions showing significant differences between individuals with ADHD and controls. No statistically significant associations between accuracy and functional connectivity were observed.

#### Conjunction analyses for the assessment of normalisation and upregulation effects

To assess whether the lack of significant case-control differences in connectivity between rSPG and left-hemispheric insula/central operculum (observed under placebo) under methylphenidate and atomoxetine reflected drug normalisation effects, we conducted confirmatory conjunction analyses. Given that the within-patient comparisons of methylphenidate/atomoxetine and placebo did not reveal any statistically significant differences across the whole brain, here we restrict our search only to voxels within the left insula/central operculum that showed abnormally high connectivity with rSPG under placebo. In the absence of a separate ADHD group, we acknowledge the potential of bias of this approach. Two conjunction analyses were performed for the comparisons of (i) controls > individuals with ADHD under placebo *and* individuals with ADHD under methylphenidate > individuals with ADHD under placebo and (ii) controls > individuals with ADHD under placebo *and* individuals with ADHD under atomoxetine > individuals with ADHD under placebo. Small volume correction using a mask defined based on the insula/central operculum cluster of case-control differences under placebo was applied to both analyses. The conjunction analyses revealed no common areas of connectivity within this cluster in individuals with ADHD under either drug and controls, thereby failing to support full normalisation of rSPG-insula/operculum hyperconnectivity.

Additional conjunction analyses were conducted to test whether regions showing heightened connectivity in ADHD under methylphenidate relative to controls during 2-back > 0-back were also upregulated with methylphenidate relative to placebo within patients. Two conjunction analyses were conducted, one for connectivity with rMFG and another one for the connectivity with rSPG. These analyses included individuals with ADHD under methylphenidate > individuals with ADHD under placebo *and* individuals with ADHD under methylphenidate > controls. Small volume correction using masks of regions showing case-control differences when ADHD individuals were under methylphenidate was applied. Conjunction analyses revealed common upregulation with methylphenidate in ADHD relative to placebo and relative to neurotypical controls in connectivity between rMFG and SMG (cluster size = 12, peak-level *Z* = 3.35, *p*_FWE_ = 0.026, MNI coordinates: 51, −36, 34) and rSPG and SMG (cluster size = 16, peak-level *Z* = 3.65, *p*_FWE_ = 0.026, MNI coordinates: 52, −36, 36). Beta values revealed that while the change between vigilance and high working memory load was similar under both placebo and methylphenidate, under methylphenidate those regions had more negative values across task conditions.

### Sensitivity analyses

Sensitivity analyses revealed no impact of oppositional defiant and/or conduct disorders on results of performance and fMRI analyses.

### Scan order effects

No scan order effects were observed for accuracy (*F*(2, 36) = 0.15, *p* = 0.858, *ƞ*^*2*^ < 0.01), MRT (*F*(2, 36) = 0.41, *p* = 0.667, *ƞ*^*2*^ < 0.01), or SDRT (*F*(2, 36) = 0.553, *p* = 0.58, *ƞ*^*2*^ = 0.01).

## Discussion

This study investigated the effects of single doses of methylphenidate and atomoxetine relative to placebo on working memory-related functional connectivity in boys with ADHD and compared to neurotypical controls. Relative to controls, individuals with ADHD under placebo exhibited increased connectivity between rSPG and left central operculum/insula. This cluster was not observed when individuals with ADHD under methylphenidate or atomoxetine were compared to controls. Under methylphenidate, youth with ADHD compared to neurotypical controls showed increased connectivity between rMFG and bilateral insula, ACC, putamen, right thalamus, and other bilateral parieto-temporal regions, as well as between rSPG and predominantly right-hemispheric ACC and parietal areas. No differences, however, were observed within patients across drug conditions or between ADHD participants under atomoxetine and controls. Participants with ADHD under each drug condition showed comparable performance to controls and no effects of drug condition on performance were observed within patients.

The hyperconnectivity in youth with ADHD relative to controls between rSPG and left central operculum/insula was no longer observed when individuals with ADHD were under either methylphenidate or atomoxetine. Combined with the lack of difference when comparing drug to placebo directly, the conjunction analysis supports a conclusion that neither medication was associated with complete functional normalisation. Furthermore, under methylphenidate, additional widespread connectivity increases were observed relative to controls between rMFG and fronto-striato-temporo-parietal regions, and between rSPG and cingulo-parietal areas. Additionally, conjunction analyses revealed that methylphenidate-related upregulation of rMFG-SMG and rSPG-SMG connectivity was observed relative to both placebo and controls. Nevertheless, the lack of differences between methylphenidate and placebo sessions across the whole brain within patients complicates the interpretation of this finding. Although our study did not observe as widespread changes in working memory-related connectivity between placebo and methylphenidate sessions as past research (Wong and Stevens 2012; Wu et al. 2017), we report for the first time methylphenidate-induced increases of working memory-related connectivity in ADHD relative to neurotypical controls. These changes were due to controls showing a negative shift in coupling between rMFG/rSPG and functionally connected regions when moving from less to more demanding conditions, while participants with ADHD under methylphenidate showing an opposite positive shift in network engagement between less and more demanding task conditions. Additionally, SMG regions showing shared upregulation of connectivity under methylphenidate relative to controls and placebo revealed similar load-dependent changes under placebo and under methylphenidate; however, methylphenidate was associated with more negative connectivity across task conditions.

The regions showing hyperconnectivity under methylphenidate relative to controls overlap with fronto-parietal networks comprising ACC, middle frontal, postcentral, supramarginal, and lingual gyri shown to be upregulated with stimulants relative to placebo in previous working memory studies in regularly treated youth with ADHD (Wong and Stevens 2012). Meta-regression analysis across fMRI studies of working memory has also reported that stimulant use was associated with increased middle frontal activation (McCarthy et al. 2014). Similarly, greater activation of insula, putamen, and ACC was associated with stimulant treatment in a meta-analysis of various cognitive paradigms (Rubia et al. 2014). Crucially, our results complement the previously published activation analysis of this dataset (Cubillo et al. 2014a), by demonstrating that methylphenidate is associated not only with increases in middle frontal, middle/superior temporal, striatal, and thalamic activation relative to controls but also with increases in the interconnectivity across these regions.

Similar to methylphenidate, atomoxetine led to a partial reduction of rSPG-operculum/insula hyperconnectivity observed in ADHD under placebo relative to controls, while not showing full normalisation or within-patients effects relative to placebo. In contrast to methylphenidate, which enhanced connectivity in several fronto-parieto-temporal and fronto-striato-thalamic regions, atomoxetine did not induce any changes relative to controls. This may be due to the single-dose design of this study. Although single doses of atomoxetine have been shown to modulate brain activation during working memory in this cohort (Cubillo et al. 2014a) and during other cognitive functions (Smith et al. 2013; Cubillo et al. 2014b; Kowalczyk et al. 2019), longer term administration is typically needed for full clinical benefits (Montoya et al. 2009), while methylphenidate shows clinical effects faster (Greenhill et al. 2001). Consequently, investigations of chronic atomoxetine treatment may be more suitable in better understanding atomoxetine's effects on neural networks in ADHD.

Our observation of abnormally increased rSPG-operculum/insula connectivity in ADHD under placebo compared to controls is consistent with past reports of hyperconnectivity in fronto-parietal networks including SPG in youth with ADHD (Massat et al. 2012; Bédard et al. 2014; Wu et al. 2017). Fronto-parietal networks are crucial to supporting maintenance and storage of information in working memory (Ekman et al. 2016) and increases in connectivity within them are associated with task engagement (Pongpipat et al. 2021) and improved task performance in neurotypical populations (Shen et al. 2015). Our finding of hyperconnectivity between rSPG and central operculum/insula in ADHD under placebo in the absence of working memory deficits may reflect a compensatory mechanism for the maintenance of working memory function, particularly considering consistent reports of widespread reductions of fronto-striatal and temporo-parietal activation in ADHD during working memory (Silk et al. 2005; Vance et al. 2007; Kobel et al. 2009; Cortese et al. 2012; Cubillo et al. 2014a; McCarthy et al. 2014; Chantiluke et al. 2015), including in the same cohort (Cubillo et al. 2014a), and during other executive functions (Cortese et al. 2012; Hart et al. 2013; Norman et al. 2016; Rubia 2018; Lukito et al. 2020). The differences were due to controls showing a negative shift in rSPG connectivity when switching between baseline vigilance and high working memory load, while patients showed similar network engagement in both conditions. The decreases of connectivity with increasing task load in neurotypical individuals were unexpected given the frequent reports of load-dependent increases of activation and connectivity in task-relevant networks (O’Hare et al. 2008; van den Bosch et al. 2014; Vogan et al. 2016; Le et al. 2020) and given an association between higher task load and greater activation in task-relevant regions in our control sample (Supplementary Materials; Cubillo et al. 2014a). This may be due to ongoing maturation processes occurring in adolescence. In fact, greater load-dependent changes are present in adults than children (Vogan et al. 2016) and increasing age has been associated with decreased working memory-related engagement of superior frontal, postcentral, inferior parietal, and cingulate regions in adolescence (Andre et al. 2015). Consequently, the reductions of connectivity with increasing load in our control group may reflect the progressive specialisation of higher order regions through weakened connections of diffuse networks. Conversely, individuals with ADHD under placebo did not show load-dependent changes, potentially indicating less efficient adjustment to changing task demands in ADHD. This could have been tested by investigating the 3-back condition; however, we opted against it due to the large error rate confounding this comparison and increasing variance within blocks, thus reducing sensitivity to group differences. Furthermore, greater parietal connectivity in ADHD relative to neurotypical peers may relate to functional maturation delay of working memory networks (Andre et al. 2015) given evidence that younger children exhibit less load-dependent modulation of task-relevant networks (van den Bosch et al. 2014). This could suggest that connectivity differences between age-matched neurotypical youth and youth with ADHD are similar to a comparison between older and younger children. This would be in line with resting state connectivity studies showing maturation lag in the connections between fronto-parietal and attention networks and the default mode network in ADHD (Sripada et al. 2014). Finally, some of these findings may also be attributed to sex differences, given that our sample comprised only boys and males with ADHD tend to exhibit more pronounced differences from their neurotypical peers than females with ADHD (Valera et al. 2010; Dupont et al. 2022).

Lack of drug effects, case-control differences, or main effects of working memory load within controls on rDLPFC connectivity were unexpected. However, while this region was chosen due to its strong involvement in verbal n-back tasks, this seed was derived from a meta-analysis in adults (Owen et al. 2005). The functional maturation processes occurring in childhood and adolescence involve progressive shifting from diffuse to localised and specialised networks (Casey et al. 2005; Rubia 2013; Andre et al. 2015; Stevens 2016). Given that DLPFC is among the last regions to mature (Casey et al. 2005; Rubia 2013), lack of drug or working memory load effects on functional connectivity with rDLPFC might reflect the young age of our cohort. Although rDLPFC showed task-relevant activation during high working memory load relative to vigilance in our control group, it may be that the functional connections between rDLPFC and other task-relevant regions have not yet fully matured, and thus did not show the expected task-dependent modulation or case-control differences and drug effects.

Lack of methylphenidate and atomoxetine effects on task performance may be due to the absence of working memory deficits observed in our ADHD cohort. Although there is meta-analytic evidence for working memory performance deficits in ADHD, they seem larger in visuospatial than verbal domain (Martinussen et al. 2005; Willcutt et al. 2005; Kasper et al. 2012). Selection of a verbal n-back task was motivated by its wide use in neuroimaging (Owen et al. 2005; Yaple and Arsalidou 2018), facilitating comparisons with previous studies. Furthermore, neurofunctional differences between individuals with ADHD and controls, despite similar performance, have been seen in previous studies using n-back (Massat et al. 2012; Cubillo et al. 2014a; Wu et al. 2017) and other verbal working memory tasks (Hale et al. 2007; Wolf et al. 2009). While the interpretation of functional implications of drug-related modulation of brain networks in these cases is not straightforward, the lack of performance differences ensures that the observed neurofunctional changes are task-specific rather than a reflection of error monitoring. Nonetheless, future research should consider using visuospatial working memory tasks in investigations of medication effects given evidence that stimulants lead to greater improvements in the visuospatial domain (Bédard and Tannock 2008; Bédard et al. 2014). Furthermore, we cannot rule out that lack of performance differences was due to low sample size, given that this study was powered for fMRI analyses (Thirion et al. 2007).

This study is strengthened by a double-blind, placebo-controlled design and inclusion of medication-naïve individuals with ADHD. Considering previous reports of associations between long-term stimulant treatment and structural (Nakao et al. 2011; Frodl and Skokauskas 2012; Lukito et al. 2020), neurochemical (Fusar-Poli et al. 2012), and neurofunctional changes in ADHD (Konrad et al. 2007; Hart et al. 2013; McCarthy et al. 2014; Norman et al. 2016; Lukito et al. 2020), investigating drug effects without the confounds of previous pharmacotherapy is critical.

This study is not without limitations. Our participants were unmedicated at the time of the study randomisation potentially because many of them were newly diagnosed with ADHD and have not yet begun medication treatment. While recruitment of a medication-naïve cohort protected against the confounding impact of chronic pharmacological treatment, it may have biased our sample towards those with less severe symptoms (Hong et al. 2014). Nonetheless, this is unlikely given that the scores of our ADHD cohort on the SDQ questionnaire were above the clinical threshold for ADHD and similar to those typically reported in the ADHD literature (Becker et al. 2006; Hall et al. 2019). While the single-dose design avoided the influence of symptomatic improvement, side effects, or neural changes associated with long-term treatment, it likely favoured detection of methylphenidate effects. Methylphenidate offers immediate clinical benefits (Greenhill et al. 2001), while atomoxetine requires longer term administration (Montoya et al. 2009). Furthermore, although the within-patient design minimised the influence of between-subject variability on drug effects, it meant that patients were only truly medication-naïve on their first visit. There is, however, no evidence suggesting that a single dose of methylphenidate or atomoxetine can lead to lasting neural changes; furthermore, to minimise any carry-over effects, study visits were scheduled one week apart, corresponding to more than five half-lives of each medication (Dhariwal and Jackson 2003). The within-patient design also meant that patients completed the task three times, while controls only once, for ethical and financial reasons. Although the n-back task is associated with improved performance after repeated practice (Pergher et al. 2018; Chen et al. 2019), the lack of scan order effects in this study indicates it is unlikely the current findings are confounded by practice effects. Furthermore, conjunction analyses could have benefited either from two independent cohorts, to first define case-control differences and then separately test drug effects, or from a baseline scan for patients. Such data, however, was not available, and thus the placebo condition appears in both contrasts. Therefore, this analysis is not completely without bias. Consequently, the reliability of these findings should be investigated in a well-powered replication study. The greater prevalence of ADHD in boys meant that this study recruited a fully male sample. Consequently, given the sexual dimorphism of ADHD and evidence of distinct neurofunctional profiles in males and females with ADHD, the generalisability of the current findings is limited (Valera et al. 2010; Dupont et al. 2022). Finally, to maximise cohort homogeneity, we only recruited right-handed individuals with combined ADHD, meaning these findings may not generalise to other ADHD populations, such as adults, left-handed individuals, or those with inattentive or hyperactive-impulsive presentations.

Overall, this is the first study of comparative effects of methylphenidate and atomoxetine on working memory-related functional connectivity in medication-naïve boys with ADHD. We showed that while both drugs shared incomplete normalising effect of abnormally enhanced connectivity in parieto-insular regions, only methylphenidate, but not atomoxetine, led to widespread increases in connectivity within task-relevant networks in ADHD relative to controls. This evidence extends past research showing upregulating effects of methylphenidate on functional connectivity during working memory in ADHD (Wong and Stevens 2012; Wu et al. 2017), suggesting they are exclusive to methylphenidate. Furthermore, in the context of past research exploring the comparative effects of single-dose methylphenidate and atomoxetine (Cubillo et al. 2014a), this study suggests that while both drugs show shared upregulation of working memory-relevant activation and a down-modulatory effect on functional connectivity abnormalities present under placebo, indicative of network reorganisation, only methylphenidate leads to widespread connectivity increases in fronto-temporo-parietal and fronto-striato-thalamic regions.

## Supplementary information

ESM 1 (DOCX 1529 kb)

## Data Availability

This is a secondary data analysis. The participants of the original study did not give written consent for their data to be shared publicly, therefore raw study data is not available.

## References

[CR1] American Psychiatric Association (2013). Diagnostic and statistical manual of mental disorders.

[CR2] Andre J, Picchioni M, Zhang R, Toulopoulou T (2015). Working memory circuit as a function of increasing age in healthy adolescence: a systematic review and meta-analyses. NeuroImage Clin.

[CR3] Ashburner J (2007). A fast diffeomorphic image registration algorithm. NeuroImage.

[CR4] Becker A, Steinhausen H-C, Baldursson G (2006). Psychopathological screening of children with ADHD: strengths and difficulties questionnaire in a pan-European study. Eur Child Adolesc Psychiatry.

[CR5] Bédard A-CV, Newcorn JH, Clerkin SM (2014). Reduced prefrontal efficiency for visuospatial working memory in attention-deficit/hyperactivity disorder. J Am Acad Child Adolesc Psychiatry.

[CR6] Bédard A-CV, Tannock R (2008). Anxiety, methylphenidate response, and working memory in children with ADHD. J Atten Disord.

[CR7] Brett M, Anton J-L, Valabregue R, Poline J-B (2002). Region of interest analysis using an SPM toolbox. NeuroImage.

[CR8] Bymaster FP, Katner JS, Nelson DL (2002). Atomoxetine increases extracellular levels of norepinephrine and dopamine in prefrontal cortex of rat: a potential mechanism for efficacy in Attention Deficit/Hyperactivity Disorder. Neuropsychopharmacology.

[CR9] Casey BJ, Tottenham N, Liston C, Durston S (2005). Imaging the developing brain: what have we learned about cognitive development?. Trends Cogn Sci.

[CR10] Chan YPM, Swanson JM, Soldin SS (1983). Methylphenidate hydrochloride given with or before breakfast: II. Effects on plasma concentration of methylphenidate and ritalinic acid. Pediatrics.

[CR11] Chantiluke K, Barrett N, Giampietro V (2015). Disorder-dissociated effects of fluoxetine on brain function of working memory in attention deficit hyperactivity disorder and autism spectrum disorder. Psychol Med.

[CR12] Chen C-C, Kuo J-C, Wang W-J (2019). Distinguishing the visual working memory training and practice effects by the effective connectivity during n-back tasks: a DCM of ERP study. Front Behav Neurosci.

[CR13] Coghill DR, Seth S, Pedroso S (2014). Effects of methylphenidate on cognitive functions in children and adolescents with attention-deficit/hyperactivity disorder: evidence from a systematic review and a meta-analysis. Biol Psychiatry.

[CR14] Coghill DR, Toplak M, Rhodes S, Adamo N (2018). Cognitive functioning in ADHD: inhibition, memory, temporal discounting, decision-making, timing and reaction time variability. Oxford Textbook of Attention Deficit Hyperactivity Disorder.

[CR15] Conners CK (2008). Conners parent rating scale, 3rd Editio.

[CR16] Cortese S, Adamo N, Del Giovane C (2018). Comparative efficacy and tolerability of medications for attention-deficit hyperactivity disorder in children, adolescents, and adults: a systematic review and network meta-analysis. Lancet Psychiatry.

[CR17] Cortese S, Kelly C, Chabernaud C (2012). Toward systems neuroscience of ADHD: a meta-analysis of 55 fMRI studies. Am J Psychiatry.

[CR18] Cubillo AI, Smith AB, Barrett N (2014). Drug-specific laterality effects on frontal lobe activation of atomoxetine and methylphenidate in attention deficit hyperactivity disorder boys during working memory. Psychol Med.

[CR19] Cubillo AI, Smith AB, Barrett N (2014). Shared and drug-specific effects of atomoxetine and methylphenidate on inhibitory brain dysfunction in medication-naive ADHD boys. Cereb Cortex.

[CR20] Dhariwal K, Jackson A (2003). Effect of length of sampling schedule and washout interval on magnitude of drug carryover from period 1 to period 2 in two-period, two-treatment bioequivalence studies and its attendant effects on determination of bioequivalence. Biopharm Drug Dispos.

[CR21] Dupont G, van Rooij D, Buitelaar JK (2022). Sex-related differences in adult attention-deficit hyperactivity disorder patients - an analysis of external globus pallidus functional connectivity in resting-state functional MRI. Front Psychiatry.

[CR22] Ekman M, Fiebach CJ, Melzer C (2016). Different roles of direct and indirect frontoparietal pathways for individual working memory capacity. J Neurosci.

[CR23] Faraone SV (2018). The pharmacology of amphetamine and methylphenidate: relevance to the neurobiology of attention-deficit/hyperactivity disorder and other psychiatric comorbidities. Neurosci Biobehav Rev.

[CR24] Friston KJ, Williams S, Howard R (1996). Movement-related effects in fMRI time-series. Magn Reson Med.

[CR25] Frodl T, Skokauskas N (2012). Meta-analysis of structural MRI studies in children and adults with attention deficit hyperactivity disorder indicates treatment effects. Acta Psychiatr Scand.

[CR26] Fusar-Poli P, Rubia K, Rossi G (2012). Striatal dopamine transporter alterations in ADHD: Pathophysiology or adaptation to psychostimulants? A meta-analysis. Am J Psychiatry.

[CR27] Gallezot J-D, Weinzimmer D, Nabulsi N (2011). Evaluation of [^11^C]MRB for assessment of occupancy of norepinephrine transporters: studies with atomoxetine in non-human primates. NeuroImage.

[CR28] Gau SS-F, Shang C-Y (2010). Improvement of executive functions in boys with attention deficit hyperactivity disorder: an open-label follow-up study with once-daily atomoxetine. Int J Neuropsychopharmacol.

[CR29] Ginestet CE, Simmons A (2011). Statistical parametric network analysis of functional connectivity dynamics during a working memory task. NeuroImage.

[CR30] Goldberg DP, Murray RM (2002). The Maudsley handbook of practical psychiatry.

[CR31] Goodman R, Scott S (1999). Comparing the strengths and difficulties questionnaire and the child behavior checklist: is small beautiful?. J Abnorm Child Psychol.

[CR32] Greenhill LL, Swanson JM, Vitiello B (2001). Impairment and deportment responses to different methylphenidate doses in children with ADHD: the MTA titration trial. J Am Acad Child Adolesc Psychiatry.

[CR33] Hale TS, Bookheimer S, McGough JJ (2007). Atypical brain activation during simple & complex levels of processing in adult ADHD: an fMRI study. J Atten Disord.

[CR34] Hall CL, Guo B, Valentine AZ (2019). The validity of the Strengths and difficulties questionnaire (SDQ) for children with ADHD symptoms. PLoS One.

[CR35] Hart H, Radua J, Nakao T (2013). Meta-analysis of functional magnetic resonance imaging studies of inhibition and attention in attention-deficit/hyperactivity disorder. JAMA Psychiatry.

[CR36] Hong J, Novick D, Treuer T (2014). Patient characteristics associated with treatment initiation among paediatric patients with attention-deficit/hyperactivity disorder symptoms in a naturalistic setting in Central Europe and East Asia. BMC Psychiatry.

[CR37] Kasper LJ, Alderson RM, Hudec KL (2012). Moderators of working memory deficits in children with attention-deficit/hyperactivity disorder (ADHD): a meta-analytic review. Clin Psychol Rev.

[CR38] Kassambara A (2020) Pipe-friendly framework for basic statistical tests [R Package]. Version 0.6.0 URL https://www.rdocumentation.org/packages/rstatix/versions/0.6.0

[CR39] Kobel M, Bechtel N, Weber P (2009). Effects of methylphenidate on working memory functioning in children with attention deficit/hyperactivity disorder. Eur J Paediatr Neurol.

[CR40] Konrad K, Neufang S, Fink GR, Herpertz-Dahlmann B (2007). Long-term effects of methylphenidate on neural networks associated with executive attention in children with ADHD: results from a longitudinal functional MRI study. J Am Acad Child Adolesc Psychiatry.

[CR41] Kowalczyk OS, Cubillo AI, Smith AB et al (2019) Methylphenidate and atomoxetine normalise fronto-parietal underactivation during sustained attention in ADHD adolescents. Eur Neuropsychopharmacol. 10.1016/j.euroneuro.2019.07.139 10.1016/j.euroneuro.2019.07.139 31358436

[CR42] Le TM, Huang AS, O’Rawe J, Leung H-C (2020) Functional neural network configuration in late childhood varies by age and cognitive state. Dev Cogn Neurosci:100862. 10.1016/j.dcn.2020.100862 10.1016/j.dcn.2020.100862 PMC7494462 32920279

[CR43] Lukito S, Norman L, Carlisi C (2020). Comparative meta-analyses of brain structural and functional abnormalities during cognitive control in attention-deficit/hyperactivity disorder and autism spectrum disorder. Psychol Med.

[CR44] Martinussen R, Hayden J, Hogg-Johnson S, Tannock R (2005). A meta-analysis of working memory impairments in children with attention-deficit/hyperactivity disorder. J Am Acad Child Adolesc Psychiatry.

[CR45] Massat I, Slama H, Kavec M et al (2012) Working memory-related functional brain patterns in never medicated children with ADHD. PLoS One 7. 10.1371/journal.pone.0049392 10.1371/journal.pone.0049392 PMC3498108 23166657

[CR46] MATLAB (2018). version 9.5.0 (R2018b).

[CR47] McCarthy H, Skokauskas N, Frodl T (2014). Identifying a consistent pattern of neural function in attention deficit hyperactivity disorder: a meta-analysis. Psychol Med.

[CR48] McLaren DG, Ries ML, Xu G, Johnson SC (2012). A generalized form of context-dependent psychophysiological interactions (gPPI): a comparison to standard approaches. NeuroImage.

[CR49] Montoya A, Hervas A, Cardo E (2009). Evaluation of atomoxetine for first-line treatment of newly diagnosed, treatment-naïve children and adolescents with attention deficit/hyperactivity disorder. Curr Med Res Opin.

[CR50] Nakao T, Radua J, Rubia K, Mataix-Cols D (2011). Gray matter volume abnormalities in ADHD: voxel-based meta-analysis exploring the effects of age and stimulant medication. Am J Psychiatry.

[CR51] National Institute for Health and Care Excellence (2008) Attention deficit hyperactivity disorder: Diagnosis and management 29634174

[CR52] Ni H-C, Shang C-Y, Gau SS-F (2013). A head-to-head randomized clinical trial of methylphenidate and atomoxetine treatment for executive function in adults with attention-deficit hyperactivity disorder. Int J Neuropsychopharmacol.

[CR53] Norman L, Carlisi C, Lukito S (2016). Structural and functional brain abnormalities in attention-deficit/hyperactivity disorder and obsessive-compulsive disorder. JAMA Psychiatry.

[CR54] O’Hare ED, Lu LH, Houston SM (2008). Neurodevelopmental changes in verbal working memory load-dependency: an fMRI investigation. NeuroImage.

[CR55] Owen AM, McMillan KM, Laird AR, Bullmore E (2005). N-back working memory paradigm: a meta-analysis of normative functional neuroimaging studies. Hum Brain Mapp.

[CR56] Penny WD, Friston KJ, Ashburner JT (2007). Statistical parametric mapping: the analysis of functional brain images.

[CR57] Pergher V, Wittevrongel B, Tournoy J (2018). N-back training and transfer effects revealed by behavioral responses and EEG. Brain Behav.

[CR58] Pfeifer JH, Allen NB (2021). Puberty initiates cascading relationships between neurodevelopmental, social, and internalizing processes across adolescence. Biol Psychiatry.

[CR59] Pievsky MA, McGrath RE (2018). The neurocognitive profile of attention-deficit/hyperactivity disorder: a review of meta-analyses. Arch Clin Neuropsychol Off J Natl Acad Neuropsychol.

[CR60] Pievsky MA, McGrath RE (2018). Neurocognitive effects of methylphenidate in adults with attention-deficit/hyperactivity disorder: a meta-analysis. Neurosci Biobehav Rev.

[CR61] Pongpipat EE, Kennedy KM, Foster CM (2021). Functional connectivity within and between n-back modulated regions: an adult lifespan psychophysiological interaction investigation. Brain Connect.

[CR62] Power JD, Barnes KA, Snyder AZ (2012). Spurious but systematic correlations in functional connectivity MRI networks arise from subject motion. NeuroImage.

[CR63] Power JD, Mitra A, Laumann TO (2014). Methods to detect, characterize, and remove motion artifact in resting state fMRI. NeuroImage.

[CR64] R Core Team (2017). R: a language and environment for statistical computing.

[CR65] Rac-Lubashevsky R, Kessler Y (2016). Decomposing the n-back task: an individual differences study using the reference-back paradigm. Mem Conscious Brain Spec Issue Honour Morris Moscovitch.

[CR66] Ramos AA, Hamdan AC, Machado L (2019) A meta-analysis on verbal working memory in children and adolescents with ADHD. Clin Neuropsychol:1–26. 10.1080/13854046.2019.1604998 10.1080/13854046.2019.1604998 31007130

[CR67] Ranganathan P, Pramesh CS, Buyse M (2015). Common pitfalls in statistical analysis: “No evidence of effect” versus “evidence of no effect”. Perspect Clin Res.

[CR68] Rommel AS, Rijsdijk F, Greven CU et al (2015) A longitudinal twin study of the direction of effects between ADHD symptoms and IQ. PLoS One 10. 10.1371/journal.pone.0124357 10.1371/journal.pone.0124357 PMC4398424 25875897

[CR69] Rubia K (2013). Functional brain imaging across development. Eur Child Adolesc Psychiatry.

[CR70] Rubia K (2018). Cognitive neuroscience of attention deficit hyperactivity disorder (ADHD) and its clinical translation. Front Hum Neurosci.

[CR71] Rubia K, Alegria AA, Cubillo AI (2014). Effects of stimulants on brain function in attention-deficit/hyperactivity disorder: a systematic review and meta-analysis. Biol Psychiatry.

[CR72] Rubia K, Halari R, Cubillo AI (2011). Methylphenidate normalizes fronto-striatal underactivation during interference inhibition in medication-naïve boys with attention-deficit hyperactivity disorder. Neuropsychopharmacology.

[CR73] Rubia K, Halari R, Mohammad A (2011). Methylphenidate normalizes frontocingulate underactivation during error processing in attention-deficit/hyperactivity disorder. Biol Psychiatry.

[CR74] Rubio Morell B, Expósito SH (2019). Differential long-term medication impact on executive function and delay aversion in ADHD. Appl Neuropsychol Child.

[CR75] Rutter M, Bailey A, Lord C (2003). Social communication questionnaire.

[CR76] Shang C-Y, Gau SS-F (2012). Improving visual memory, attention, and school function with atomoxetine in boys with attention-deficit/hyperactivity disorder. J Child Adolesc Psychopharmacol.

[CR77] Shen J, Zhang G, Yao L, Zhao X (2015). Real-time fMRI training-induced changes in regional connectivity mediating verbal working memory behavioral performance. Neuroscience.

[CR78] Sheridan MA, Hinshaw S, D’Esposito M (2010). Stimulant medication and prefrontal functional connectivity during working memory in ADHD. J Atten Disord.

[CR79] Silk T, Vance A, Rinehart N (2005). Fronto-parietal activation in attention-deficit hyperactivity disorder, combined type: functional magnetic resonance imaging study. Br J Psychiatry.

[CR80] Smith AB, Cubillo AI, Barrett N (2013). Neurofunctional effects of methylphenidate and atomoxetine in boys with attention-deficit/hyperactivity disorder during time discrimination. Biol Psychiatry.

[CR81] Sripada CS, Kessler D, Angstadt M (2014). Lag in maturation of the brain’s intrinsic functional architecture in attention-deficit/hyperactivity disorder. Proc Natl Acad Sci U S A.

[CR82] Stevens MC (2016). The contributions of resting state and task-based functional connectivity studies to our understanding of adolescent brain network maturation. Neurosci Biobehav Rev.

[CR83] Thirion B, Pinel P, Mériaux S (2007). Analysis of a large fMRI cohort: statistical and methodological issues for group analyses. NeuroImage.

[CR84] Torchiano M (2020) Efficient effect size computation [R Package]. Version 0.8.0URL https://www.rdocumentation.org/packages/effsize/versions/0.8.0

[CR85] Valera EM, Brown A, Biederman J (2010). Sex differences in the functional neuroanatomy of working memory in adults with ADHD. Am J Psychiatry.

[CR86] van den Bosch GE, Marroun HE, Schmidt MN (2014). Brain connectivity during verbal working memory in children and adolescents. Hum Brain Mapp.

[CR87] Vance A, Silk TJ, Casey M (2007). Right parietal dysfunction in children with attention deficit hyperactivity disorder, combined type: a functional MRI study. Mol Psychiatry.

[CR88] Vogan V, Morgan B, Powell T (2016). The neurodevelopmental differences of increasing verbal working memory demand in children and adults. Dev Cogn Neurosci.

[CR89] Wechsler D (1999). Wechsler abbreviated scale of intelligence WASI: manual.

[CR90] Wickham H, RStudio (2019) Easily install and load the “Tidyverse” [R Package]. Version 1.3.0URL https://tidyverse.tidyverse.org/

[CR91] Wilens TE (2008). Effects of methylphenidate on the catecholaminergic system in attention-deficit/hyperactivity disorder. J Clin Psychopharmacol.

[CR92] Willcutt EG, Doyle AE, Nigg JT (2005). Validity of the executive function theory of attention-deficit/hyperactivity disorder: a meta-analytic review. Biol Psychiatry.

[CR93] Witcher JW, Long A, Smith B (2003). Atomoxetine pharmacokinetics in children and adolescents with attention deficit hyperactivity disorder. J Child Adolesc Psychopharmacol.

[CR94] Wolf RC, Plichta MM, Sambataro F (2009). Regional brain activation changes and abnormal functional connectivity of the ventrolateral prefrontal cortex during working memory processing in adults with attention-deficit/hyperactivity disorder. Hum Brain Mapp.

[CR95] Wong CG, Stevens MC (2012). The effects of stimulant medication on working memory functional connectivity in attention-deficit/hyperactivity disorder. Biol Psychiatry.

[CR96] Wu Z-M, Bralten J, An L (2017). Verbal working memory-related functional connectivity alterations in boys with attention-deficit/hyperactivity disorder and the effects of methylphenidate. J Psychopharmacol (Oxf).

[CR97] Yang L, Cao Q, Shuai L (2012). Comparative study of OROS-MPH and atomoxetine on executive function improvement in ADHD: a randomized controlled trial. Int J Neuropsychopharmacol.

[CR98] Yaple Z, Arsalidou M (2018). N-back working memory task: meta-analysis of normative fMRI studies with children. Child Dev.

