# Supplementary material for: Single-dose effects of methylphenidate and atomoxetine on functional connectivity during an n-back task in boys with ADHD
Source: Psychopharmacology (Berl). 2023 Jul 27;240(10):2045–60. doi: 10.1007/s00213-023-06422-7 (PMC10506949; doi:10.1007/s00213-023-06422-7)
Supplement: Supplementary file 1 — (DOCX 1529 kb) [file 213_2023_6422_MOESM1_ESM.docx]

***Supplementary Materials for***

**Single-dose effects of methylphenidate and atomoxetine on functional connectivity during an n-back task in boys with ADHD**

**Table of contents:**

[1. Analyses assessing working memory networks involved in the n-back task 2](#_Toc95227988)

[1.1. Methods 2](#_Toc95227989)

[1.2. Results 2](#_Toc95227990)

[2. References 8](#_Toc95227991)

#

# Analyses assessing working memory networks involved in the n-back task

## Methods

To assess activation, statistical modelling was performed using general linear models (GLMs). Individual regressors were created for each task condition, including instruction, 0-, 1-, 2-, and 3-back trials. All task regressors were convolved with a canonical haemodynamic response function. Additionally, 24 motion parameters estimated according to Friston et al. (1996) together with scrubbing regressors for volumes exceeding framewise displacement of 0.5mm (Power et al. 2012, 2014) were included.

To assess the quality of the GLMs and to confirm that the task robustly elicited activation in established regions involved in working memory networks reported in the literature, a one-sample *t*-test of the contrast exploring high working memory load relative to the baseline vigilance condition (2-back > 0-back) was conducted in the neurotypical control group. This is a classic working memory contrast studied in the n-back task and thus has been chosen for this analysis(Owen et al. 2005). Further, we decided against using the 3-back condition for this comparison due to poor accuracy in the control group (67.4%). Cluster-based family-wise error (FWE) correction (*p* < 0.05) with a cluster-forming threshold of *p*_uncorrected_ < 0.001 was applied to account for multiple comparisons.

## Results

As an initial step, we confirmed that the n-back task produced the expected activation in regions associated with working memory in the neurotypical control group. In line with past literature (Owen et al. 2005; Andre et al. 2015; Yaple and Arsalidou 2018), high working memory load (2-back > 0-back) was associated with activation in bilateral superior parietal/lateral occipital, middle and superior frontal gyri, insula, frontal pole, anterior cingulate/paracingulate, as well as right precuneus (Figure S2; Table S2).

**Figure S1.** Overlap between the chosen seed regions (shown in blue) and activation during high working memory load (2-back > 0-back; shown in red-yellow) in the neurotypical control group. Axial slices are marked with the *z* coordinate reflecting distance in millimetres from the anterior-posterior commissure.
DLPFC = Dorsolateral Prefrontal Cortex; MFG = Middle Frontal Gyrus; SPG = Superior Parietal Gyrus.


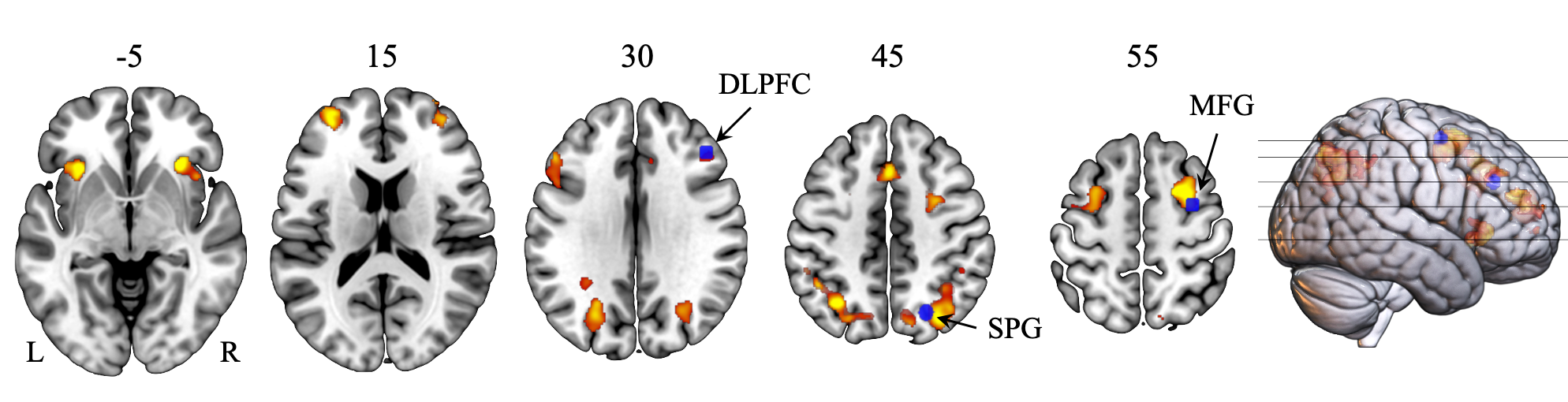


**Table S1.** Significant clusters of activation in neurotypical controls during the 2-back > 0-back contrast

| **Cluster** | **Cerebral Region** | **Cluster Size (voxels)** | **Peak MNI Coordinate** | | | **Peak *z*** | **Cluster *p_FWE_*** |
| --- | --- | --- | --- | --- | --- | --- | --- |
|  |  |  | **x** | **y** | **z** |  |  |
| 1 | L Lateral Occipital | 3124 | -26 | -66 | 36 | 5.28 | <0.001 |
|  | L Lateral Occipital/Superior Parietal |  | -32 | -60 | 45 | 4.74 |  |
|  | L Supramarginal |  | -42 | -46 | 39 | 4.73 |  |
| 2 | R Lateral Occipital | 2937 | 28 | -62 | 34 | 4.62 | <0.001 |
|  | R Lateral Occipital |  | 30 | -69 | 45 | 4.62 |  |
|  | R Precuneus |  | 9 | -64 | 42 | 4.25 |  |
| 3 | R Middle Frontal | 1566 | 28 | 2 | 50 | 5.2 | <0.001 |
|  | R Superior Frontal |  | 22 | 8 | 58 | 5.03 |  |
| 4 | L Paracingulate | 1193 | -10 | 21 | 38 | 5.11 | <0.001 |
|  | L Paracingulate/Anterior Cingulate |  | 3 | 20 | 40 | 4.84 |  |
|  | R Paracingulate |  | 9 | 33 | 38 | 4.59 |  |
| 5 | L Middle/Superior Frontal | 1018 | -24 | 6 | 50 | 4.75 | <0.001 |
|  | L Middle Frontal |  | -28 | -3 | 57 | 4.52 |  |
|  | L Middle Frontal |  | -28 | 10 | 57 | 4.25 |  |
| 6 | L Frontal Pole | 985 | -32 | 50 | 15 | 4.64 | <0.001 |
|  | L Frontal Pole |  | -39 | 52 | 0 | 3.82 |  |
| 7 | R Insula | 882 | 30 | 22 | -4 | 5.01 | <0.001 |
|  | R Insula |  | 40 | 16 | -4 | 4.11 |  |
|  | R White Matter |  | 27 | 20 | 12 | 3.23 |  |
| 8 | R Frontal Pole | 860 | 40 | 44 | 21 | 4.73 | <0.001 |
|  | R Frontal Pole |  | 38 | 51 | 15 | 4.27 |  |
|  | R Frontal Pole |  | 32 | 63 | 15 | 4.26 |  |
| 9 | L Middle Frontal | 809 | -50 | 27 | 33 | 4.65 | <0.001 |
|  | L Middle Frontal |  | -44 | 22 | 27 | 4.15 |  |
|  | L Middle Frontal |  | -52 | 20 | 33 | 3.96 |  |
| 10 | L Insula | 706 | -33 | 18 | -8 | 5.17 | 0.001 |
|  | L Insula |  | -28 | 22 | 3 | 4.44 |  |
| 11 | R Frontal Pole | 394 | 44 | 38 | 36 | 4.04 | 0.018 |
|  | R Middle Frontal |  | 45 | 27 | 32 | 3.71 |  |
|  | R Middle Frontal |  | 38 | 27 | 28 | 3.56 |  |

FWE = Family-wise Error; L = Left; MNI = Montreal Neurological Institute; R = Right.

Subsequently, we confirmed that the gPPI model was able to detect context-dependent modulation of functional connectivity estimates. Neurotypical controls showed greater functional connectivity during the baseline vigilance condition compared to high working memory load (0-back > 2-back) between the right MFG and bilateral parietal operculum, supramarginal, occipital fusiform, and temporal occipital regions, the right insula and inferior temporal gyrus, as well as the brainstem. Greater connectivity during 0-back > 2-back has also been observed between the right SPG and bilateral insula and central operculum, left lateral occipital cortex, as well as right parietal operculum and Heschl's gyrus/planum polare (Figure S2; Table S2). No effect of connectivity has been observed for the right DLPFC.

**Figure S2.** Regions showing significant clusters of functional connectivity with the right MFG (shown in yellow) and with the right SPG (shown in red) during 0-back > 2-back in the neurotypical control group. Axial slices are marked with the *z* coordinate reflecting distance in millimetres from the anterior-posterior commissure.
MFG = Middle Frontal Gyrus; SPG = Superior Parietal Gyrus.


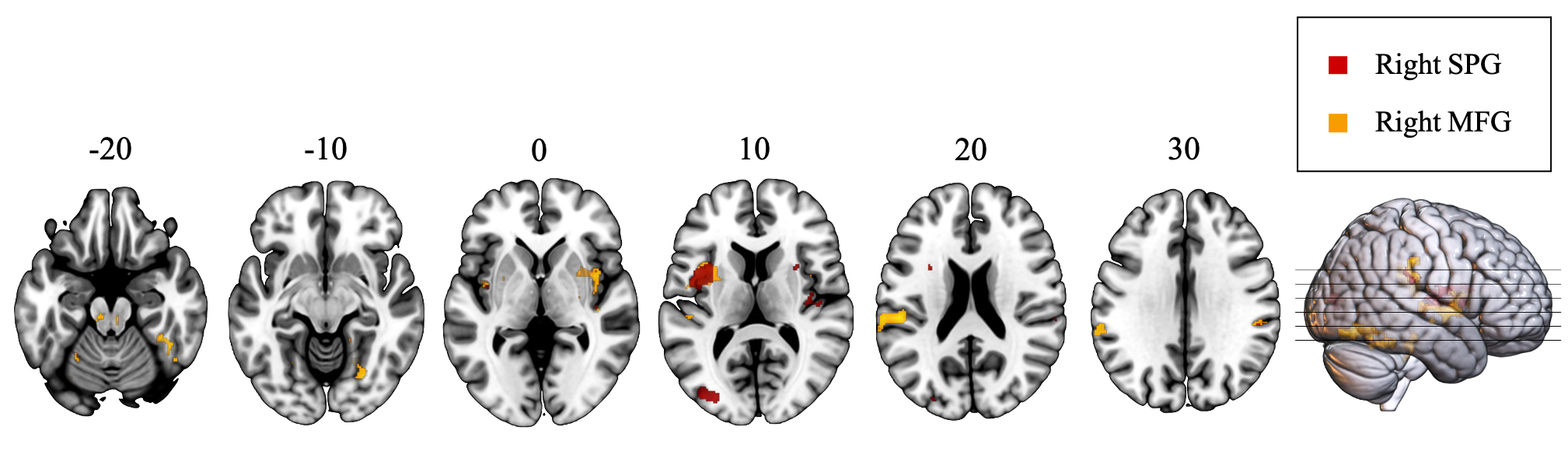


**Table S2.** Significant clusters of functional connectivity in neurotypical controls during the 0-back > 2-back contrast.

| **Cluster** | **Cerebral Region** | **Cluster Size (voxels)** | **Peak MNI Coordinate** | | | **Peak *z*** | **Cluster *p_FWE_*** |
| --- | --- | --- | --- | --- | --- | --- | --- |
|  |  |  | **x** | **y** | **z** |  |  |
| **MFG Connectivity During 0-back > 2-back** | | | | | | | |
| 1 | L Parietal Operculum | 1489 | -50 | -27 | 20 | 4.35 | <0.001 |
|  | L Parietal Operculum |  | -50 | -38 | 27 | 4.19 |  |
|  | L Supramarginal |  | -63 | -28 | 24 | 4.08 |  |
| 2 | L Central Operculum | 1335 | -42 | -2 | 14 | 4.29 | <0.001 |
|  | L Frontal Operculum |  | -36 | 10 | 12 | 4.24 |  |
|  | L Central Operculum |  | -42 | 8 | 8 | 3.75 |  |
| 3 | R Planum Polare/Heschl’s Gyrus | 983 | 42 | -21 | 2 | 3.93 | 0.001 |
|  | R Insula |  | 40 | 3 | 0 | 3.91 |  |
|  | R Insula |  | 40 | -12 | 2 | 3.67 |  |
| 4 | R Occipital Fusiform | 749 | 30 | -70 | -15 | 4.33 | 0.003 |
|  | R Occipital Fusiform |  | 26 | -78 | -15 | 4.02 |  |
|  | R Lingual |  | 20 | -56 | -14 | 3.71 |  |
| 5 | R Parietal Operculum | 701 | 56 | -27 | 24 | 4.39 | 0.004 |
|  | R Supramarginal |  | 60 | -30 | 34 | 4.08 |  |
| 6 | L Occipital Fusiform | 667 | -34 | -72 | -16 | 4.58 | 0.005 |
|  | L Cerebellum |  | -24 | -56 | -24 | 3.59 |  |
|  | L Temporal Occipital Fusiform |  | -32 | -62 | -15 | 3.56 |  |
| 7 | R Temporal Occipital Fusiform | 497 | 39 | -45 | -22 | 3.93 | 0.021 |
|  | R Temporal Occipital Fusiform/Inferior Temporal |  | 46 | -57 | -20 | 3.78 |  |
|  | R Inferior Temporal |  | 48 | -54 | -12 | 3.61 |  |
| 8 | Brainstem | 468 | -10 | -28 | -18 | 3.88 | 0.027 |
|  | Brainstem |  | 2 | -28 | -24 | 3.64 |  |
|  | Brainstem |  | 9 | -32 | -34 | 3.49 |  |
| **SPG Connectivity during 0-back > 2-back** | | | | | | | |
| 1 | L Central Operculum | 1463 | -36 | -2 | 18 | 4.55 | <0.001 |
|  | L Insula |  | -34 | -10 | 15 | 3.91 |  |
|  | L White Matter |  | -30 | 12 | 18 | 3.91 |  |
| 2 | R Heschl’s Gyrus/Planum Polare | 779 | 40 | -20 | 2 | 3.79 | 0.001 |
|  | R Insula |  | 36 | -3 | 16 | 3.78 |  |
|  | R Insula |  | 38 | -16 | 10 | 3.58 |  |
| 3 | L Lateral Occipital | 518 | -38 | -84 | 9 | 3.72 | 0.011 |
|  | L Lateral Occipital |  | -28 | -84 | 9 | 3.71 |  |
|  | L Lateral Occipital |  | -27 | -86 | 20 | 3.46 |  |
| 4 | R Parietal Operculum | 446 | 46 | -27 | 24 | 3.98 | 0.021 |
|  | R Parietal Operculum |  | 57 | -27 | 22 | 3.83 |  |
|  | R Parietal/Central Operculum |  | 40 | -20 | 20 | 3.12 |  |

FWE = Family-wise Error; L = Left; MFG = Middle Frontal Gyrus; MNI = Montreal Neurological Institute; R = Right; SPG = Superior Parietal Gyrus.

# References

Andre J, Picchioni M, Zhang R, Toulopoulou T (2015) Working memory circuit as a function of increasing age in healthy adolescence: A systematic review and meta-analyses. NeuroImage Clin 12:940–948. https://doi.org/10.1016/j.nicl.2015.12.002

Friston KJ, Williams S, Howard R, et al (1996) Movement-related effects in fMRI time-series. Magn Reson Med 35:346–355. https://doi.org/10.1002/mrm.1910350312

Owen AM, McMillan KM, Laird AR, Bullmore E (2005) N-back working memory paradigm: A meta-analysis of normative functional neuroimaging studies. Hum Brain Mapp 25:46–59. https://doi.org/10.1002/hbm.20131

Power JD, Barnes KA, Snyder AZ, et al (2012) Spurious but systematic correlations in functional connectivity MRI networks arise from subject motion. NeuroImage 59:2142–2154. https://doi.org/10.1016/j.neuroimage.2011.10.018

Power JD, Mitra A, Laumann TO, et al (2014) Methods to detect, characterize, and remove motion artifact in resting state fMRI. NeuroImage 84:320–341. https://doi.org/10.1016/j.neuroimage.2013.08.048

Yaple Z, Arsalidou M (2018) N-back working memory task: Meta-analysis of normative fMRI studies with children. Child Dev 89:2010–2022. https://doi.org/10.1111/cdev.13080
